# Non-canonical heterogeneous cellular distribution and co-localization of CaMKIIα and CaMKIIβ in the spinal superficial dorsal horn

**DOI:** 10.1007/s00429-017-1566-0

**Published:** 2017-11-18

**Authors:** Max Larsson

**Affiliations:** Department of Clinical and Experimental Medicine, Division of Neurobiology, Linköping University, SE-581 85 Linköping, Sweden

**Keywords:** Pain, Central sensitization, Spinal cord, Pax2, GABA

## Abstract

Ca^2+^/calmodulin-dependent protein kinase II (CaMKII) is a key enzyme in long-term plasticity in many neurons, including in the nociceptive circuitry of the spinal dorsal horn. However, although the role of CaMKII heterooligomers in neuronal plasticity is isoform-dependent, the distribution and co-localization of CaMKII isoforms in the dorsal horn have not been comprehensively investigated. Here, quantitative immunofluorescence analysis was used to examine the distribution of the two major neuronal CaMKII isoforms, α and β, in laminae I–III of the rat dorsal horn, with reference to inhibitory interneurons and neuronal populations defined by expression of parvalbumin, calretinin, and calbindin D28k. Unexpectedly, all or nearly all inhibitory and excitatory neurons showed both CaMKIIα and CaMKIIβ immunoreactivity, although at highly variable levels. Lamina III neurons showed less CaMKIIα immunoreactivity than laminae I–II neurons. Whereas CaMKIIα immunoreactivity was found at nearly similar levels in inhibitory and excitatory neurons, CaMKIIβ generally showed considerably lower immunoreactivity in inhibitory neurons. Distinct populations of inhibitory calretinin neurons and excitatory parvalbumin neurons exhibited high CaMKIIα-to-CaMKIIβ immunoreactivity ratios. CaMKIIα and CaMKIIβ immunoreactivity showed positive correlation at GluA2^+^ puncta in pepsin-treated tissue. These results suggest that, unlike the forebrain, the dorsal horn is characterized by similar expression of CaMKIIα in excitatory and inhibitory neurons, whereas CaMKIIβ is less expressed in inhibitory neurons. Moreover, CaMKII isoform expression varies considerably within and between neuronal populations defined by laminar location, calcium-binding protein expression, and transmitter phenotype, suggesting differences in CaMKII function both between and within neuronal populations in the superficial dorsal horn.

## Introduction

Ca^2+^/calmodulin-dependent protein kinase II (CaMKII) is well established as a pivotal enzyme in neuronal plasticity (Hell 2014; Lisman et al. 2012; Coultrap and Bayer 2012). CaMKII exists as heterooligomers of α, β, γ, and δ isoforms, of which α and β are essentially restricted to neurons, whereas γ and δ are more widely expressed. Most studies on the role of CaMKII in neuronal plasticity have focused on the α subunit or used tools that do not differentiate between different isoforms. For instance, autophosphorylation of CaMKIIα at T286 has been shown to be critical for learning and long-term potentiation (LTP) at some glutamatergic synapses (Hell 2014; Lisman et al. 2012). Nevertheless, major functional differences between CaMKIIα and CaMKIIβ have been identified (Liu and Murray 2012; Hell 2014). The β isoform binds F-actin and targets α/β heteromers to dendritic spines (e.g., Borgesius et al. 2011; Shen et al. 1998); this binding is abolished by Ca^2+^/calmodulin or autophosphorylation, allowing the holoenzyme to translocate to the postsynaptic density in an activity-dependent manner (Shen and Meyer 1999). Moreover, CaMKIIβ is more sensitive than CaMKIIα to Ca^2+^/calmodulin and Ca^2+^ spike frequency, whereas heteromers show intermediate sensitivity (Brocke et al. 1999; De Koninck and Schulman 1998). The subunit composition of CaMKII oligomers in a cell is stochastically determined based on the relative expression of each isoform (Shen et al. 1998). Thus, differential expression patterns of CaMKII isoforms confer cell-wide differences in functional characteristics of the enzyme between neuronal populations. Notably, with some exceptions such as cerebellar Purkinje cells, CaMKIIα has been reported to be essentially restricted to excitatory neurons in the rodent CNS (Benson et al. 1992; Sík et al. 1998). The cell-specific distribution of CaMKIIβ is less characterized, although at least some populations of inhibitory neuron express this isoform (Ochiishi et al. 1994; Burgin et al. 1990).

Similar to its role in the brain, CaMKII has been strongly implicated in spinal sensory plasticity, including primary afferent long-term potentiation, hyperalgesia, and sensitization of dorsal horn neurons to sensory stimuli (e.g., Fang et al. 2002; Yang et al. 2004; Zeitz et al. 2004; Larsson and Broman 2006; Larsson 2009; Larsson and Broman 2011). However, the role of CaMKII in spinal nociceptive plasticity remains enigmatic and, in some instances, appears counter to established model mechanisms of CaMKII-mediated neuronal plasticity. For instance, some types of spinal plasticity and hyperalgesia appear to not require CaMKII activation, or autophosphorylation of CaMKIIα (Jones and Sorkin 2005; Zeitz et al. 2004). Furthermore, we have observed, in the capsaicin model of hyperalgesia, a curious downregulation of CaMKII and autophosphorylated CaMKII in the postsynaptic density of non-peptidergic C fiber synapses; this downregulation is concomitant with upregulation of GluA1-containing α-amino-3-hydroxy-5-methyl-4-isoxazole propionic acid (AMPA) receptors at the same synapses (Larsson and Broman 2006, 2008). It is possible that this discordance with respect to the established model of CaMKII-mediated synaptic plasticity is attributed to differential isoform expression and function of CaMKII between dorsal horn neurons. As a first step to investigate this issue, the present study was devoted to examine the distribution and co-localization of CaMKIIα and CaMKIIβ in certain excitatory and inhibitory neuronal populations in the superficial dorsal horn.

## Materials and methods

### Tissue preparation

Adult male Sprague–Dawley rats were anaesthetized with sodium pentobarbital (60 mg, i.p.) and rapidly perfused transcardially with phosphate-buffered saline (PBS, 300 mOsm, pH 7.4) followed by PBS containing 4% paraformaldehyde (0.5–1 L, 20 min). After perfusion, the lumbar spinal cord (L3–L5) was removed and placed in 30% sucrose in PBS. In addition, spinal cord tissue perfusion fixed with 4% paraformaldehyde was obtained from 4-week-old mice deficient in CaMKIIα (C*amk2a* ^−/−^) (Elgersma et al. 2002) or CaMKIIβ (C*amk2b* ^−/−^) (Gao et al. 2014) and corresponding wild-type mice (a gift from Y. Elgersma). Transverse spinal cord sections were cut on a freezing microtome at a thickness of 40 µm and stored at − 20 °C in cryoprotectant (30% glycerol and 30% ethylene glycol in 0.1 M phosphate buffer, pH 7.4) until use. All animal experiments were approved by the local Research Animal Care and Use Committee.

### Antibodies

Primary antibodies used in this study are specified in Table 1. The mouse anti-CaMKIIα clone 6G9 (Erondu and Kennedy 1985) has been widely used and its selectivity for CaMKIIα is well characterized. For instance, very little staining was observed in the early postnatal rat cerebral cortex (Ding et al. 2013), where CaMKIIβ but not CaMKIIα is expressed at considerable levels (Burgin et al. 1990). The mouse anti-CaMKIIβ antibody produced no staining in the brain of *Camk2b* ^−/−^ mice (Bachstetter et al. 2014; van Woerden et al. 2009). The guinea pig anti-NeuN antibody recognizes the NeuN/Fox3 protein and produced very similar pattern of staining in the rat spinal cord as the original NeuN antibody (Todd et al. 1998; Larsson 2017). Similarly, guinea pig antibodies directed towards calbindin D28k, calretinin, and parvalbumin yielded immunolabeling patterns in the rat spinal cord and brain consistent with the previous reports (e.g., Antal et al. 1991; Ren and Ruda 1994; Antal et al. 1990). The Pax2 antibody used here has been shown to specifically and selectively label essentially all GABAergic neurons in the spinal dorsal horn of adult rats (Larsson 2017). The GluA2 antibody used in combination with pepsin-mediated antigen retrieval has been shown to label synaptic GluA2 (Polgár et al. 2008).

**Table 1:** Primary antibodies used

Antigen	Host, isotype	Clone	Immunogen	Supplier	Cat#	Lot#	Concentration
Calbindin D28k	Guinea pig	Polyclonal	Human aa 3–251	Synaptic Systems	214 004	214004/5	1:250
Calretinin	Guinea pig	Polyclonal	Mouse protein	Synaptic Systems	214 104	214104/2	1:500
CaMKIIα	Mouse, IgG_1_	6G9	Rat protein	Millipore	MAB8699	21020859	1:1000
CaMKIIα	Mouse, IgG_1_	6G9	Rat protein	Thermo Fisher	MA1-048	QI222632	1:1000
CaMKIIβ	Mouse, IgG_2b_	CB-beta-1	Rat protein	LifeSpan	LS-B5767	60012, 69070	1:200
GluA2	Mouse, IgG_2a_	6C4	N-terminal	Millipore	MAB397	2049209	1:250
Iba1	Rabbit	EPR16588	Mouse aa 100–147	Abcam	Ab178846	GR249899	1:1000
NeuN	Guinea pig	Polyclonal	Mouse aa 1–97	Synaptic Systems	266 004	266004/5	1:250
Parvalbumin	Guinea pig	Polyclonal	Rat protein	Synaptic Systems	195 004	195004/9	1:500
Pax2	Rabbit	Polyclonal	Human aa 268–332	Atlas Antibodies	HPA047704	R44792	1:50–1:100

### Immunofluorescence

As the CaMKIIα and CaMKIIβ, mouse antibodies were of different subclasses (IgG_1_ and IgG_2b_, respectively), it was possible to use subclass-specific secondary antibodies for simultaneous detection of the isoform on the same spinal cord sections. Moreover, in most cases, quadruple immunofluorescent labeling was performed using the CaMKII antibodies in combination with rabbit anti-Pax2 and a guinea pig antibody for NeuN or a calcium-binding protein. In some cases, mouse tissue sections were subjected to heat-induced antigen retrieval using a citrate buffer (pH 6.1; DAKO, Glostrup, Denmark) at 97 °C for 20 min. Briefly, lumbar spinal cord sections were incubated in PBS containing 3% normal goat serum, 0.5% bovine serum albumin and 0.5% Triton X-100 (blocking solution) and in primary antibodies diluted in blocking solution (see Table 1) overnight or for 2 days (for calbindin and calretinin immunofluorescence, to allow antibody penetration throughout the section) at room temperature. After washing in PBS, the sections were incubated in goat anti-rabbit Alexa Fluor 405 (1:250) or donkey anti-rabbit Brilliant Violet 421 (1:100), goat anti-mouse IgG_2b_ Alexa Fluor 488 (1:500), goat anti-mouse IgG_1_ Alexa Fluor 568 (1:500), and goat anti-guinea pig Alexa Fluor 647 (1:500) for 2–4 h. Sections were mounted on glass slides and coverslipped using Prolong Gold (Life Technologies). In one set of experiments, CaMKIIα and CaMKIIβ antibodies were mixed with biotinylated isolectin B4 (1:500; Life Technologies), which was detected using streptavidin-Alexa Fluor 405 (1:250; Life Technologies). In one experiment, to enable labeling of CaMKII in the postsynaptic density, rat tissue sections were subject to antigen retrieval by incubation in pepsin (DAKO; 1 mg/mL in 10 mM HCl) at 37 °C for 7 min prior to the immunofluorescence procedure (Larsson et al. 2013; Nagy et al. 2004; Polgár et al. 2008; Watanabe et al. 1998). In this case, anti-GluA2 was used as an excitatory synaptic marker. To test the specificity of the subclass-specific anti-mouse secondary antibodies, sections were incubated with either CaMKIIα or CaMKIIβ antibody and subsequently in both goat anti-mouse IgG_2b_ Alexa Fluor 488 and goat anti-mouse IgG_1_ Alexa Fluor 568; no cross-reactivity for the other IgG subclass was evident for either secondary antibody (see also Manning et al. 2012). In one NeuN/Pax2 double labeling experiment, goat anti-rabbit Alexa Fluor 488 (1:500) and goat anti-guinea pig Alexa Fluor 568 (1:500) were used for secondary detection. All secondary antibodies were from Life Technologies, except donkey anti-rabbit Brilliant Violet 421, which was from BioLegend (San Diego, CA, USA).

### Microscopy

To compare the laminar distribution of CaMKIIα and CaMKIIβ immunolabeling, an Olympus BX51 microscope was used to acquire epifluorescence and dark field micrographs with a 10×/0.3 objective. For all other microscopy, a Zeiss LSM700 confocal microscope was used. For quantitative analysis, z-stacks of optical sections at 1 µm separation were acquired of the entire superficial dorsal horn (laminae I–III) throughout the thickness of the tissue section using the automatic tile scan function with a 40×/1.3 oil immersion objective. To limit bleaching and acquisition time, pixel dwell time and frame averaging were set to minimal values, while pixel width was set to 96 nm. For each fluorescence channel, the same gain and offset were used for all sections from an animal in a given experiment. In the case of the tissue treated with pepsin to reveal synaptic proteins, a 63×/1.4 oil immersion objective was used to acquire single optical slices of 400 µm^2^ regions (pixel size 40 nm) of laminae I–III at the surface of the section, where GluA2 immunopositive puncta were evident. Micrographs for publication were acquired using the 63×/1.4 oil immersion objective, or, for the isolectin B4 staining experiment, as a tile scan with the 40×/1.3 oil immersion objective. Some publication micrographs were deconvolved using the Huygens software (Scientific Volume Imaging), as noted.

### Quantitative analysis

For each quadruple immunofluorescence experiment, tissue sections from two or three animals were used for quantitative analysis. The z-stack of each tissue section was opened in ImageJ and each lamina scanned for cells immunopositive for the marker of interest, while the CaMKIIα/CaMKIIβ channels were switched off. For the purpose of this study, lamina II was divided into an outer (IIo) and an inner (IIi) half of equal thickness. Care was taken to only analyze the portion of the z-stack in which the optical sections had similar overall intensities. In all experiments, immunolabeling for each antibody showed homogeneous penetration throughout the section, except occasionally at the section surface. After selection of a profile, the optical section with the largest cross section through the profile and with the most distinct profile boundaries (relative to surrounding neuropil and the profile’s nucleus) as assessed in the marker or CaMKIIα/CaMKIIβ channels was analyzed by outlining the profile border and measuring the mean intensity of the resulting region of interest in all channels. Cells where no distinct cytoplasm/nucleus boundary could be found in any optical section were discarded, as were cells for which the largest or most distinct cross section was found at either extreme optical section of the usable portion of the z-stack. To normalize channel intensities across sections and experiments, in each analyzed section, the mean channel intensities over laminae I–III were measured in one representative optical section at the center of the tissue section, and the channel intensities of each profile in that section were normalized to these average intensities. Normalized CaMKIIα and CaMKIIβ immunofluorescence intensities were very similar between sections and animals both with respect to overall immunolabeling patterns and absolute values, and results were, therefore, pooled for each experiment.

For analysis of NeuN/Pax2/CaMKIIα/CaMKIIβ labeled sections, in two animals, 50 NeuN-immunopositive cells were initially selected in each lamina of each animal to estimate the proportion of Pax2 immunopositive neurons. Subsequently, additional NeuN^+^ profiles were selected based on Pax2 immunopositivity to yield 50 NeuN^+^/Pax2^−^ and 50 NeuN^+^/Pax2^+^ cells in each lamina and animal. Cells were selected in a random manner without reference to CaMKIIα or CaMKIIβ immunolabeling; care was taken to select cells throughout the mediolateral and dorsoventral extent of each lamina. Moreover, in this experiment, ependymal cells (30 per animal) were outlined and their CaMKIIα and CaMKIIβ immunolabeling intensities measured. In an additional experiment, NeuN^+^ neurons in the superficial dorsal horn of five sections from one animal were scored from 0 to 4 with respect to CaMKIIα and CaMKIIβ immunoreactivity (where 0 represents essentially no immunoreactivity), whereas Pax2 immunoreactivity was subsequently scored as positive or negative.

In the case of parvalbumin/Pax2/CaMKIIα/CaMKIIβ labeling, all tissue sections were scanned for parvalbumin immunopositive cells with all other channels switched off. All parvalbumin cells in laminae IIi–III which fit the general criteria as outlined above were included in the analysis. In addition, to assess cytoplasmic versus nuclear labeling in parvalbumin cells, the nucleus of each parvalbumin cell was outlined (in the CaMKIIα or CaMKIIβ channel), and the area and integrated pixel density of each channel measured in the soma and nucleus. Mean cytoplasmic intensity $\bar{d}_{\text{cytoplasm}}$ was calculated using the following equation:

$$\bar{d}_{\text{cytoplasm}} = \frac{{D_{\text{soma}} - D_{\text{nucleus}} }}{{A_{\text{soma}} - A_{\text{nucleus}} }},$$

where *D*_soma_ and *D*_nucleus_ are the integrated density of the soma and nucleus, respectively, and *A*_soma_ and *A*_nucleus_ are the areas of these compartments.

In calretinin/Pax2/CaMKIIα/CaMKIIβ labeled sections, analysis was conducted in laminae I–II, where all calretinin immunolabeled cells that fitted the general criteria were included in the analysis. In sections immunolabeled for calbindin D28k/Pax2/CaMKIIα/CaMKIIβ, the initial scanning suggested that few calbindin D28k cells expressed Pax2. Therefore, the quantitative analysis was restricted to Pax2^−^ cells. One hundred calbindin D28k^+^/Pax2^−^ cells were randomly selected for analysis in each lamina and animal.

For analysis of synaptic CaMKII, 200 GluA2 immunopositive puncta were randomly selected in each lamina in micrographs of pepsin-treated tissue. The puncta were outlined and the intensities of CaMKIIα and CaMKIIβ immunofluorescence measured.

For statistical comparison of groups, one-way or two-way ANOVA followed by Tukey’s post hoc test, or Kruskal–Wallis test followed by Dunn’s multiple comparison test was used as applicable. For correlations, Spearman’s correlation was used.

## Results

### Validation of isoform-specific CaMKII antibodies

**Fig. 1:**
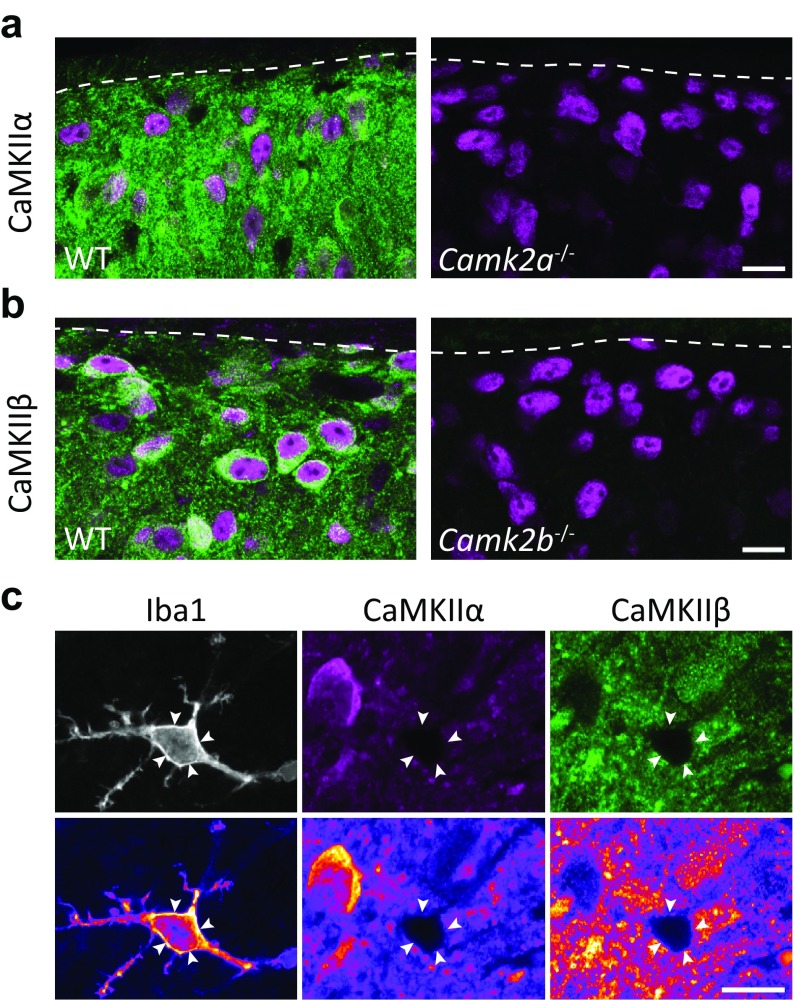
Validation of CaMKIIα and CaMKIIβ antibodies for immunofluorescence in the spinal dorsal horn. **a** CaMKIIα (green) and NeuN (magenta) immunofluorescence in the superficial dorsal horn of wild-type and CaMKIIα^−/−^ mice. CaMKIIα immunolabeling was essentially absent in CaMKIIα-deficient mice. **b** CaMKIIβ (green) and NeuN (magenta) immunofluorescence in the superficial dorsal horn of wild-type and CaMKIIβ^−/−^ mice. CaMKIIβ immunolabeling was essentially absent from CaMKIIβ^−/−^ mice, apart from a weak staining of myelin. Dashed lines in **a** and **b** indicate the dorsal border of lamina I. Micrographs are single optical sections obtained using a 63×/1.4 objective. Scale bar, 10 µm, valid for **a** and **b**. **c** Example of an Iba1^+^ microglial cell in lamina II of the rat spinal cord devoid of both CaMKIIα and CaMKIIβ immunoreactivity. Bottom panels are pseudocolored to better visualize weak immunolabeling. Arrowheads indicate the microglial cell body. Micrographs are singe optical sections obtained with a 63×/1.4 objective. Scale bar, 10 µm

Although the CaMKII antibodies used have been well characterized and shown to be specific for the respective isoform in the brain (e.g., Bachstetter et al. 2014; Ding et al. 2013; van Woerden et al. 2009), antibody specificity tests should preferentially be performed on the tissue of interest, as antigen cross-reactivity may be observed in only certain types or regions of tissue (Larsson et al. 2011). Therefore, spinal cord tissue from mice in which each isoform had been genetically ablated was used to validate the specificity and selectivity of the antibodies also in the spinal cord. Whereas wild-type mice showed strong CaMKIIα-like immunoreactivity (CaMKIIα-LI) in the superficial dorsal horn (as well as somewhat weaker and more scattered labeling of cell bodies and processes in deeper laminae), spinal cord sections from *Camk2a* ^−/−^ mice were devoid of immunolabeling (Fig. 1a). CaMKIIβ-like immunoreactivity (CaMKIIβ-LI) was weak in wild-type mouse spinal cord sections, possibly because the tissue was too strongly fixed, and heat-mediated antigen retrieval was, therefore, used. This yielded a pattern of CaMKIIβ-LI in wild-type mouse spinal cord similar to that in rat spinal cord not subjected to antigen retrieval (Fig. 1b; see below); however, an additional weak immunolabeling over myelin was observed (not shown). No CaMKIIβ-LI was evident in cell bodies or neuropil in spinal cord from *Camk2b* ^−/−^ mice, although some myelin-associated immunolabeling was observed also in this tissue. In rat spinal cord, neither CaMKIIα-LI nor CaMKIIβ-LI were observed in Iba1^+^ microglial cell bodies (Fig. 1c), further confirming the specificity of both CaMKII antibodies in the rodent spinal cord.

### General distribution of CaMKIIα and β in the dorsal horn

**Fig. 2:**
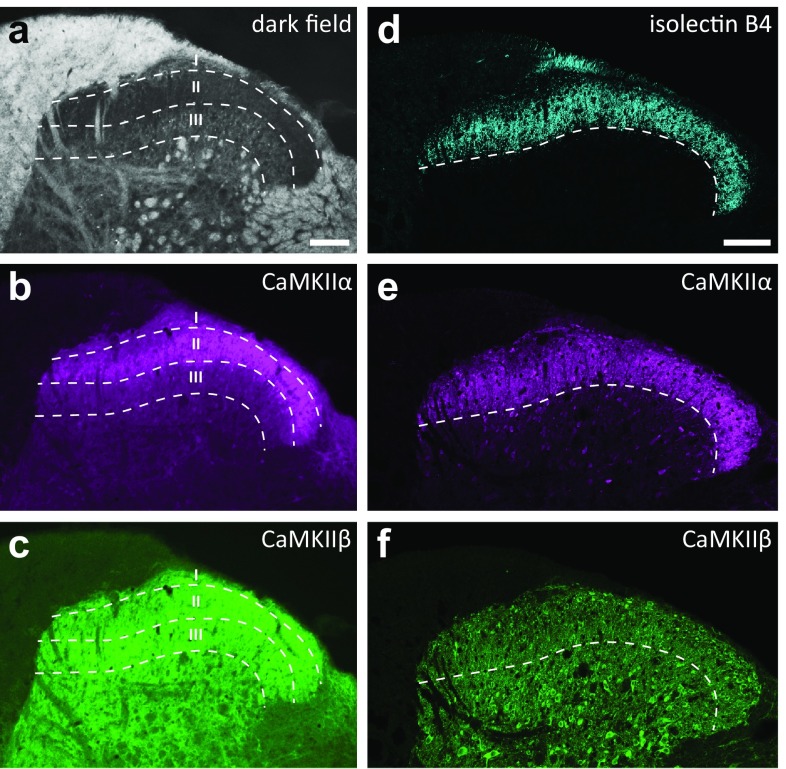
Laminar distribution of CaMKIIα-LI and CaMKIIβ-LI in the lumbar dorsal horn. Dark field microscopy (**a**) combined with epifluorescence of CaMKIIα-LI (**b**) and CaMKIIβ-LI (**c**) shows enrichment of CaMKIIα-LI but not CaMKIIβ-LI in laminae I–II. Borders between laminae I–III are indicated by dashed lines. Confocal microscopy of isolectin B4 binding (**d**) and CaMKIIα-LI (**e**) and CaMKIIβ-LI (**f**) imaged using a ×40 objective and tile scan. Note the correspondence between the ventral limit of isolectin B4 binding and the ventral aspect of the enrichment CaMKIIα-LI in lamina II (indicated by dashed lines). Scale bars in **a** and **d** are 100 µm, valid for **a**–**c** and **d**–**f**, respectively

As described previously (Benson et al. 1992; Brüggemann et al. 2000; Terashima et al. 1994), CaMKIIα-LI was highly enriched in the neuropil of laminae I–II of the rat lumbar dorsal horn (Fig. 2b, e), although in the medial spinal cord, lamina IIo was somewhat less strongly labeled than the inner part of this lamina. Lamina III showed lower levels of immunoreactivity, whereas even weaker immunoreactivity was found in deeper laminae. The ventral limit of the neuropil enrichment of CaMKIIα-LI coincided with the border between laminae II and III, as assessed using dark field microscopy (Fig. 2a, b) and isolectin B4 binding (Fig. 2c, d). Nevertheless, despite weaker overall immunolabeling, cell bodies showing substantial immunoreactivity for CaMKIIα were abundant also in lamina III and in deeper laminae of the dorsal horn (cf. Terashima et al. 1994). In some cell bodies, the nucleus (except the nucleolus) was prominently immunolabeled, whereas other cells had only very weakly immunolabeled nuclei. However, in a given cell, the nucleus was nearly always less strongly immunolabeled for CaMKIIα than the cytoplasm. In the neuropil, some thick processes were outlined by immunoreactivity for CaMKIIα; some of these were strongly labeled, whereas others were more weakly labeled than the surrounding tissue.

**Fig. 3:**
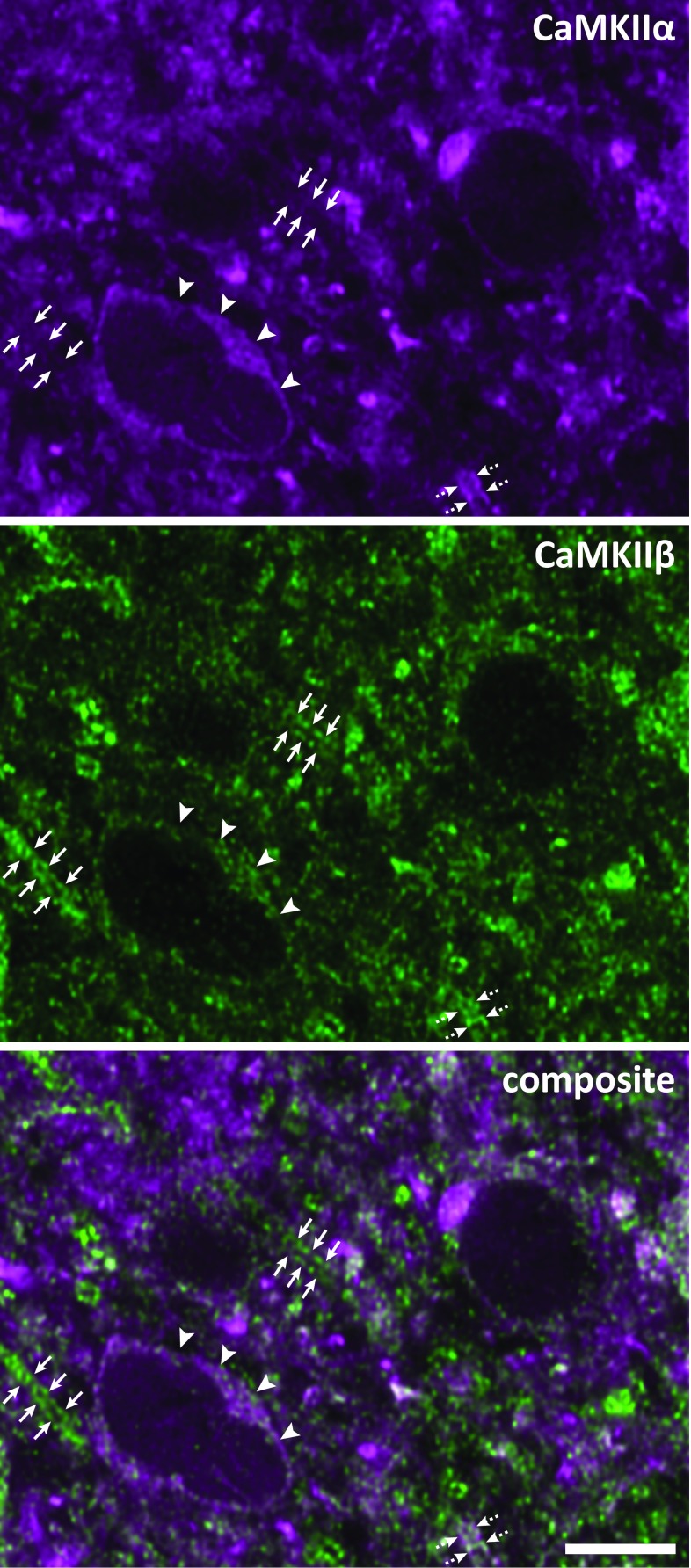
CaMKIIα-LI and CaMKIIβ-LI in neuropil and cell bodies in lamina II. Shown is a high-magnification view of a portion of lamina IIi, exemplifying the differential patterns of immunolabeling for CaMKIIα and CaMKIIβ in the superficial dorsal horn. The micrograph is a single deconvolved optical section. CaMKIIα-LI is relatively diffused and concentrated in the somatic cytoplasm and as larger puncta in the neuropil, whereas CaMKIIβ-LI is more fine-grained, both in somata and neuropil. Arrowheads indicate a neuron which shows moderate cytoplasmic staining for both CaMKIIα and CaMKIIβ. Note, however, the very weak CaMKIIβ-LI in the nucleus. Arrows indicate two processes with strong CaMKIIβ-LI but weak CaMKIIα-LI, whereas dashed arrows indicate a process segment exhibiting strong CaMKIIα-LI as well as CaMKIIβ-LI. Scale bar is 5 µm

In contrast to CaMKIIα, CaMKIIβ-like immunoreactivity (CaMKIIβ-LI) in the dorsal horn was relatively evenly distributed throughout the dorsal horn (Fig. 2c, f). Apart from a slightly weaker labeling in medial lamina IIo, there was no visually discernible difference in either neuropil or somatic staining between different laminae. Although the strength of cytoplasmic CaMKIIβ-LI varied substantially between neuronal cell bodies, most nuclei showed very weak or undetectable staining. CaMKIIβ-LI had a fine granular appearance both in neuropil and somata, whereas CaMKIIα-LI was more diffuse and concentrated in larger puncta or processes (Fig. 3). Furthermore, considering the supposed co-assembly of CaMKIIα and CaMKIIβ into heterooligomers, the overlap in the neuropil between CaMKIIα-LI and CaMKIIβ-LI was relatively incomplete. For instance, whereas some elongated processes co-localized substantial CaMKIIα-LI and CaMKIIβ-LI, other such processes with strong CaMKIIβ-LI showed poor labeling for CaMKIIα (Fig. 3) and vice versa (not shown).

### CaMKII isoforms in excitatory and inhibitory neurons

**Fig. 4:**
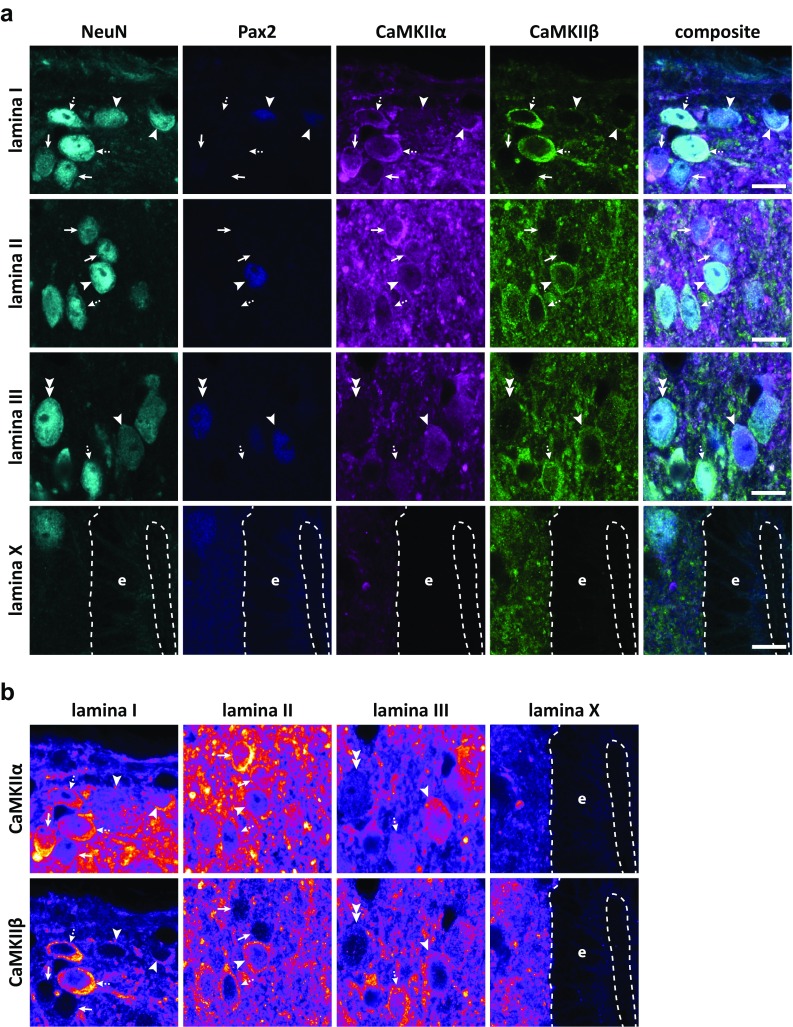
CaMKIIα-LI and CaMKIIβ-LI in excitatory and inhibitory dorsal horn neurons. **a** Examples of NeuN immunolabeled cells in laminae I–III that differentially label for Pax2, CaMKIIα and CaMKIIβ. Arrowheads indicate Pax2^+^ cells with varying CaMKIIα-LI and CaMKIIβ-LI. Arrows indicate Pax2^−^ cells with strong CaMKIIα-LI and weak CaMKIIβ-LI, while dashed arrows indicate Pax2^−^ cells with both strong CaMKIIα-LI and strong CaMKIIβ-LI. Double arrowhead indicates a Pax2^+^ cell that shows both weak CaMKIIα-LI and weak CaMKIIβ-LI. Note the variable nuclear CaMKIIα-LI and consistently weak nuclear CaMKIIβ-LI. Also note the substantial CaMKIIα-LI also in some lamina III cells. Shown is also a micrograph of lamina X and ependymal cells (e) lining the central canal. Dashed lines indicate basal and apical borders of the ependymal cell layer. Note the very weak CaMKIIα-LI and CaMKIIβ-LI over ependymal cells. **b** Same panels of CaMKIIα-LI and CaMKIIβ-LI as shown in a, but using a false-color look-up table to increase the visibility of weak immunofluorescence. Note that cells with weak CaMKIIα-LI or CaMKIIβ-LI, nevertheless, show substantially stronger immunolabeling as compared to the ependymal cells. Scale bars are 10 µm, valid for all panels

**Fig. 5:**
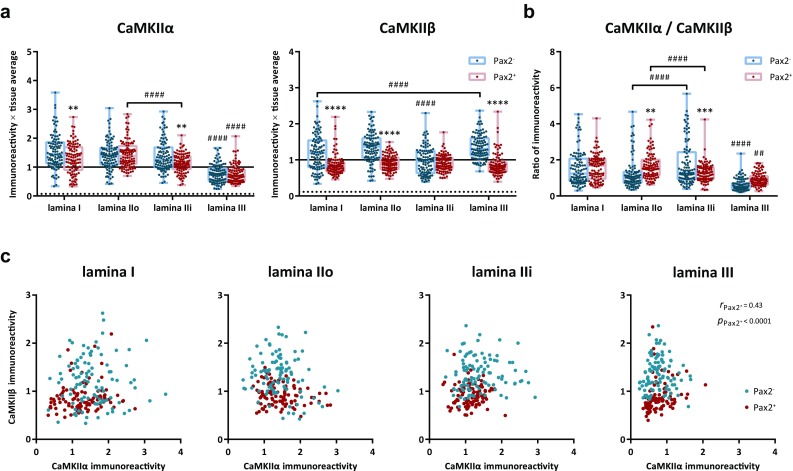
Quantitative analysis of CaMKIIα-LI and CaMKIIβ-LI in excitatory and inhibitory dorsal horn neurons. **a** CaMKIIα-LI and CaMKIIβ-LI normalized against the average intensity over laminae I–III in Pax2^−^ and Pax2^+^ neurons in laminae I–III. Solid horizontal lines indicate average tissue labeling over laminae I–III. Dashed lines indicate average immunolabeling over ependymal cells lining the central canal. **b** Ratio of normalized CaMKIIα-LI over normalized CaMKIIβ-LI in Pax2^−^ and Pax2^+^ neurons in laminae I–III. Asterisks in **a** and **b** indicate statistical comparison between Pax2^−^ and Pax2^+^ cells within the same lamina, whereas hashes indicate statistical comparisons between laminae within either the Pax2^−^ or the Pax2^+^ group. **^/##^ *p* < 0.01; ***p < 0.001; ****^/####^ *p* < 0.0001; two-way ANOVA followed by Tukey’s post hoc test. **c** scatterplots of normalized CaMKIIβ-LI versus normalized CaMKIIα-LI in each lamina. Correlations were assessed using Spearman’s correlation. Only Pax2^+^ neurons in lamina III were weak correlation detected

An immunofluorescence protocol was established to co-immunolabel for CaMKIIα and CaMKIIβ together with NeuN as a pan-neuronal marker and Pax2 as a marker for inhibitory neurons in the same spinal cord sections (Fig. 4). In an initial selection of NeuN^+^ cells, Pax2 immunolabeling was detected in 26, 29, 33, and 18% of cells in laminae I, IIo, IIi, and III, respectively. Additional cells were then analyzed to yield 100 each of Pax2^+^ and Pax2^−^ cells in each lamina. In laminae I–III, somatic labeling for CaMKIIα varied widely between neurons; some showed strong immunolabeling, whereas in others, the immunolabeling was barely detectable. Similarly, somatic CaMKIIβ-LI varied between neurons from very weak to very strong. Notably, ependymal cells lining the central canal showed essentially no immunolabeling for CaMKIIα or CaMKIIβ, suggesting that even the very weak immunolabeling of some neurons reflected the presence of the respective isoform rather than constituting unspecific labeling of cellular elements. Indeed, quantitative analysis showed that the most weakly immunolabeled neurons were more than threefold more strongly labeled for either isoform than were ependymal cells, whose normalized CaMKIIα-LI and CaMKIIβ-LI were 0.07 ± 0.009 (mean ± SD; *n* = 60 cells) and 0.11 ± 0.03 times tissue average, respectively (Fig. 5a).

Further quantitative analysis showed that Pax2^−^ (presumed excitatory) neurons in lamina I, dorsal lamina II (IIo) and ventral lamina II (IIi) had similar levels of CaMKIIα-LI, whereas lamina III Pax2^−^ neurons were on average considerably less immunolabeled (Fig. 5a). Similarly, among Pax2^+^ (presumed inhibitory) neurons, those in laminae I–II were on average more strongly immunolabeled for CaMKIIα than were those in lamina III. Notably, Pax2^+^ neurons showed similar or only slightly weaker CaMKIIα-LI than Pax2^−^ neurons in the same lamina. For CaMKIIβ-LI, among Pax2^−^ neurons, those in lamina IIi showed weaker average immunolabeling than those in other laminae. However, Pax2^+^ neurons generally exhibited considerably weaker CaMKIIβ-LI than Pax2^−^ neurons in all laminae except for lamina IIi, where the lack of difference could be attributed to the weak labeling also in Pax2^−^ neurons. Notably, a similar pattern of CaMKIIα-LI and CaMKIIβ-LI between Pax2^−^ and Pax2^+^ neurons in different laminae was found in an additional experiment analyzed using manual scoring (Tables 2, 3).

**Table 2:** CaMKIIα-LI in Pax2^−^ and Pax2^+^ neurons analyzed by manual scoring

Score	Lamina							
	I		IIo		IIi		III	
	Pax2^−^ (136) (%)	Pax2^+^ (32) (%)	Pax2^−^ (188) (%)	Pax2^+^ (72) (%)	Pax2^−^ (104) (%)	Pax2^+^ (82) (%)	Pax2^−^ (75) (%)	Pax2^+^ (59) (%)
0	0	1.7	0	0	0	0	1.3	8.5
1	11.0	24.6	12.8	15.3	14.4	31.7	66.7	54.2
2	53.5	45.6	52.7	70.8	65.4	64.6	30.7	35.6
3	27.5	28.0	29.8	13.9	18.3	3.7	1.3	1.7
4	8.0	0	4.8	0	1.9	0	0	0
Statistical significance					*			

Number of neurons analyzed in each group is indicated in parentheses. *p < 0.05; Kruskal–Wallis test followed by Dunn’s post hoc test (within-lamina comparisons)

**Table 3:** CaMKIIβ-LI in Pax2^−^ and Pax2^+^ neurons analyzed by manual scoring

Score	Lamina							
	I		IIo		IIi		III	
	Pax2^−^ (168) (%)	Pax2^+^ (47) (%)	Pax2^−^ (188) (%)	Pax2^+^ (72) (%)	Pax2^−^ (104) (%)	Pax2^+^ (82) (%)	Pax2^−^ (75) (%)	Pax2^+^ (59) (%)
0	0	0	0	0	0	0	0	0
1	8.0	17.5	16.5	20.8	25.0	28.0	9.3	37.3
2	48.5	68.4	23.9	58.3	47.1	68.3	40.0	45.8
3	29.5	14.0	46.8	20.8	27.9	3.7	49.3	16.9
4	14.0	0	12.8	0	0	0	1.3	0
Statistical significance	*		****				****	

Number of neurons analyzed in each group is indicated in parentheses. *p < 0.05; **p < 0.01; ****p < 0.0001; Kruskal–Wallis test followed by Dunn’s post hoc test (within-lamina comparisons)

As the subunit composition of CaMKII heteromers is stochastically determined on the basis of the relative expression of the different isoforms (Shen et al. 1998), it is of interest to assess the ratio of CaMKIIα-LI to CaMKIIβ-LI. Although the absolute ratio of expression could not be determined (as the immunolabeling efficiency for each isoform was unknown), it was possible to semi-quantitatively compare the ratio of immunolabeling between different profiles. Surprisingly, in both lamina IIo and lamina III, Pax2^+^ neurons had a higher CaMKIIα-LI-to-CaMKIIβ-LI ratio than Pax2^−^ neurons (Fig. 5b). In lamina IIi, the mean ratio was similar between Pax2^+^ and Pax2^−^ neurons, but among the latter, a subpopulation showed a high ratio of CaMKIIα-LI to CaMKIIβ-LI. Scatter plots revealed no or only weak correlation between CaMKIIα-LI and CaMKIIβ-LI within different neuronal populations (Fig. 5c).

Whereas the proportion of Pax2^+^ neurons in laminae I–II were in accordance with the frequency of inhibitory neurons in these laminae, the proportion of Pax2^+^ cells in lamina III was lower than expected (Polgár et al. 2003; Todd and Sullivan 1990). However, for detection of Pax2 immunolabeling, a secondary antibody conjugated to Alexa Fluor 405 was used, which resulted in relatively weak immunofluorescence. In a separate NeuN/Pax2 double labeling experiment where Pax2 immunofluorescence was detected using Alexa Fluor 568, many NeuN^+^ cells, in particular in lamina III, were only weakly labeled for Pax2; such cells may have been undetected when using the Alexa Fluor 405 fluorophore. Indeed, 23 of 50 NeuN^+^ cells (46%) in lamina III were strongly or weakly Pax2 immunopositive in NeuN/Pax2 double immunolabeled tissue, in line with the previous estimates of the proportion of GABAergic cells in lamina III (Polgár et al. 2003; Todd and Sullivan 1990).

### CaMKII isoforms in parvalbumin neurons

Parvalbumin neurons are considered to constitute a relatively homogeneous population of inhibitory neurons in the superficial dorsal horn, although a subset of parvalbumin neurons has been reported to be non-GABAergic and thus presumably glutamatergic, at least in the rat (Antal et al. 1991; Laing et al. 1994). To determine the expression of CaMKIIα and CaMKIIβ in parvalbumin neurons of inhibitory and excitatory phenotypes, spinal cord sections were immunolabeled for the CaMKII isoforms, parvalbumin, and Pax2. Parvalbumin neurons were found both in lamina IIi, embedded in the plexus of parvalbumin processes residing in this part of the dorsal horn, and in lamina III ventral to the parvalbumin plexus. In keeping with the previous observations (Antal et al. 1991; Laing et al. 1994), a proportion of parvalbumin neurons in lamina IIi (26%; 27/102 cells) and in lamina III (42%; 53/126 cells) were found to lack Pax2 and were, therefore, assumed to be excitatory. Thus, for the purpose of this analysis, parvalbumin neurons were divided into four subpopulations, based on Pax2 expression and location in either lamina IIi or lamina III.

**Fig. 6:**
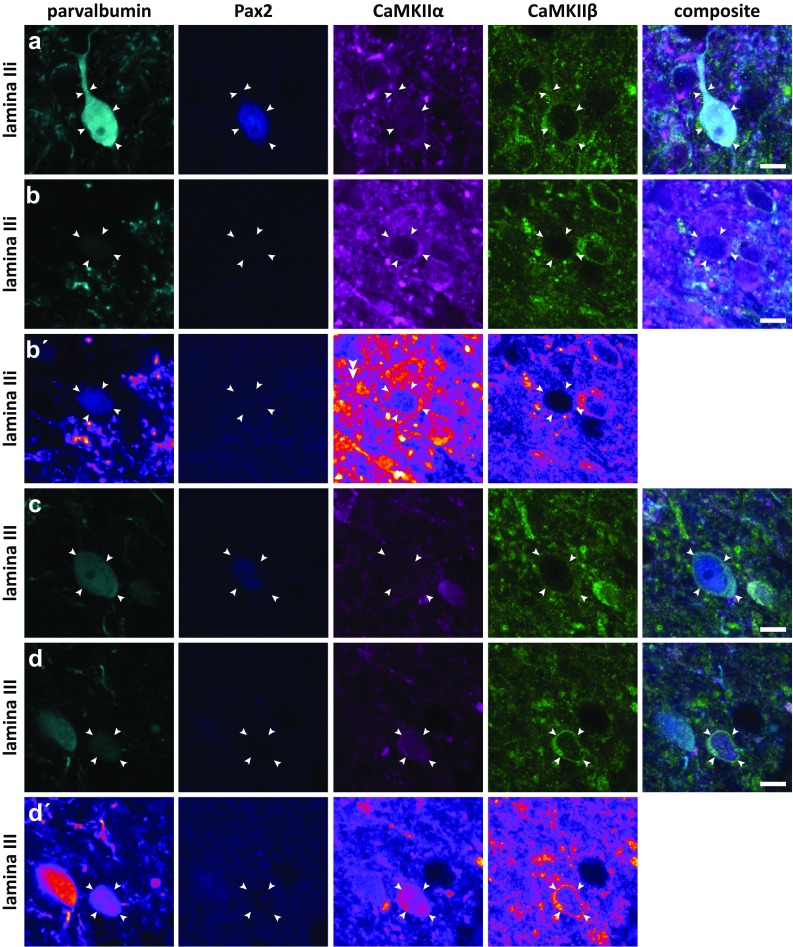
CaMKIIα-LI and CaMKIIβ-LI in parvalbumin neurons. Indicated by arrowheads are examples of parvalbumin immunolabeled cells in laminae IIi and III that differentially label for Pax2, CaMKIIα, and CaMKIIβ. **a** Pax2^+^, presumed inhibitory parvalbumin cell in lamina IIi with considerable CaMKIIα-LI and CaMKIIβ-LI. **b**, **b′** Pax2^−^, presumed excitatory, weakly parvalbumin immunopositive cell in lamina IIi exhibiting strong CaMKIIα-LI and weak CaMKIIβ-LI. In **b′**, a false-color look-up table was applied to better visualize the weak parvalbumin and cytoplasmic CaMKIIβ immunolabeling. Note the apparent lack of nuclear CaMKIIβ-LI. **c** Pax2^+^ parvalbumin neuron in lamina III showing weak CaMKIIα-LI and CaMKIIβ-LI. **d**, **d′** Pax2^−^ parvalbumin neuron in lamina III showing moderate CaMKIIα-LI and CaMKIIβ-LI. **d′** Same neuron as in d using a false-color look-up table to enhance the visibility of the parvalbumin labeling. Scale bars, 5 µm, valid for all panels

**Fig. 7:**
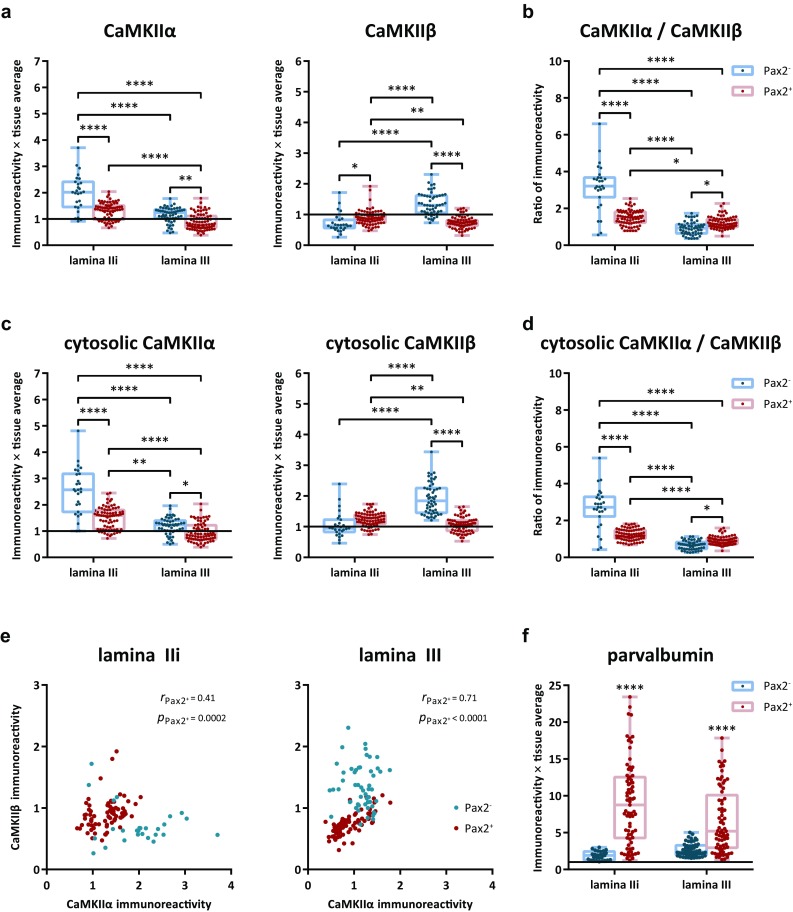
Quantitative analysis of CaMKIIα-LI and CaMKIIβ-LI in parvalbumin neurons. **a** Total somatic CaMKIIα-LI and CaMKIIβ-LI normalized against the average intensity over laminae I–III in Pax2^−^ and Pax2^+^ parvalbumin neurons in laminae IIi and III. Solid horizontal lines indicate average tissue labeling over laminae I–III. **b** Ratio of normalized CaMKIIα-LI over normalized CaMKIIβ-LI in Pax2^−^ and Pax2^+^ neurons in laminae IIi and III. **c** Cytosolic CaMKIIα-LI and CaMKIIβ-LI normalized against the average intensity over laminae I–III in Pax2^−^ and Pax2^+^ parvalbumin neurons in laminae IIi and III. Solid horizontal lines indicate average tissue labeling over laminae I–III. **d** Ratio of normalized CaMKIIα-LI over normalized CaMKIIβ-LI in the cytosol of Pax2^−^ and Pax2^+^ neurons in laminae IIi and III. *p < 0.05; **p < 0.01; ***p < 0.001; ****p < 0.0001; two-way ANOVA followed by Tukey’s post hoc test. **e** Scatterplots of normalized CaMKIIβ-LI versus normalized CaMKIIα-LI in Pax2^+^ and Pax2^−^ parvalbumin neurons in each lamina. Correlations were statistically evaluated using Spearman’s correlation. Note the strong positive correlation in Pax2^+^ neurons in lamina III, and the moderate correlation in such neurons in lamina IIi. **f** Parvalbumin immunoreactivity in Pax2^−^ and Pax2^+^ parvalbumin neurons, normalized against laminae I–III tissue average (indicated by solid line). ****p < 0.0001; two-way ANOVA followed by Sidak’s post hoc test of selected groups (Pax2^−^ versus Pax2^+^ in each lamina)

As was the case for NeuN^+^ neurons, parvalbumin neurons exhibited highly variable levels of CaMKIIα-LI and CaMKIIβ-LI (Fig. 6). Pax2^+^ neurons in either lamina IIi or III exhibited, on average, lower levels of CaMKIIα-LI than Pax2^−^ neurons in the same lamina, although neurons in lamina III had weak staining compared to those with the same Pax2 phenotype in lamina IIi (Fig. 7a). By contrast, the pattern of immunolabeling of neurons in lamina IIi and III diverged with respect to CaMKIIβ. All subpopulations showed weak immunoreactivity for this subunit, except Pax2^−^ neurons in lamina III, which exhibited comparatively strong CaMKIIβ-LI (cf. Fig. 5). Pax2^−^ neurons in lamina IIi showed a conspicuously high CaMKIIα-to-CaMKIIβ ratio as compared to the other populations (Fig. 7b).

Given that somatic CaMKIIα-LI and CaMKIIβ-LI was weaker in the nucleus as compared to the cytoplasm, it is possible that some of the measured differences in somatic immunolabeling between populations could be attributed to differences in the ratio of cytoplasm area to nucleus area between neurons. To test this for parvalbumin neurons, cytoplasmic labeling was obtained for each neuron. The patterns of cytoplasmic immunolabeling for both CaMKIIα-LI and CaMKIIβ-LI were very similar to those of total somatic labeling (Fig. 7a–d), indicating that the observed differences in immunolabeling between neuronal populations were not to a large extent attributed to differences in cytoplasm-to-nucleus ratio, at least in the case of parvalbumin neurons.

In lamina III, a strong correlation between CaMKIIβ-LI and CaMKIIα-LI was found in Pax2^+^ parvalbumin neurons; a weaker correlation was also found in Pax2^+^ neurons in lamina IIi, whereas no correlations were observed for Pax2^−^ neurons (Fig. 7e).

During the analysis of CaMKII labeling in parvalbumin neurons, it was noted that parvalbumin expression appeared related to the expression of Pax2, in that all Pax2^−^ neurons showed weak parvalbumin immunolabeling, whereas neurons with strong parvalbumin expression always were Pax2^+^. Indeed, quantitative analysis showed that Pax2^−^ neurons in both laminae IIi and III invariably had weak parvalbumin expression, whereas parvalbumin expression in Pax2^+^ neurons was highly variable (Fig. 7f).

### CaMKII isoforms in calretinin neurons

Calretinin immunolabeling in the spinal cord was as previously described (Ren and Ruda 1994). Few calretinin immunolabeled neurons were found in lamina III, and analysis was, therefore, constrained to laminae I–II. Some reports have suggested that a small population of calretinin neurons are GABAergic in the mouse dorsal horn (Huang et al. 2010; Smith et al. 2015) and in isolated embryonic rat dorsal horn neurons (Albuquerque et al. 1999). However, to my knowledge, this issue has not been investigated in the intact rat spinal cord. Thus, spinal cord sections immunolabeled for calretinin, Pax2, CaMKIIα, and CaMKIIβ were specifically examined with respect to calretinin/Pax2 co-localization. Eight percent (7/86), 12% (28/235), and 5% (11/226) of calretinin neurons were immunopositive for Pax2 in lamina I, lamina IIo, and lamina IIi, respectively. Thus, a minor fraction of calretinin neurons are presumably GABAergic also in the rat dorsal horn.

**Fig. 8:**
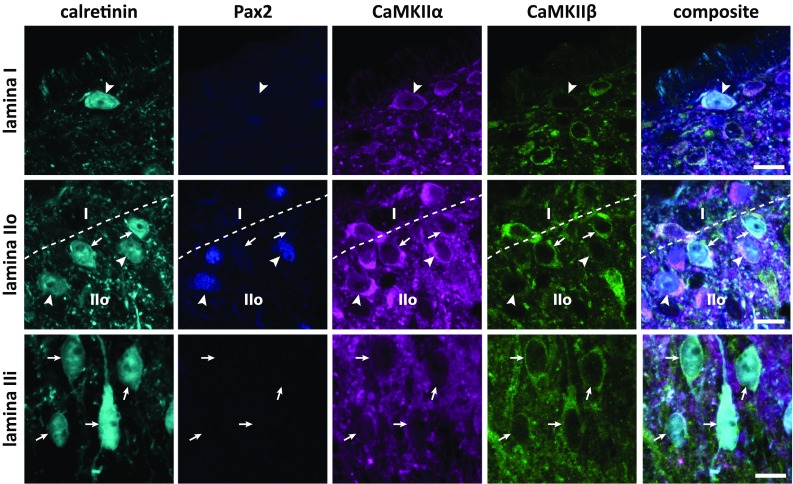
CaMKIIα-LI and CaMKIIβ-LI in calretinin neurons. Indicated are examples of calretinin immunolabeled cells in laminae I–IIi that differentially label for Pax2, CaMKIIα, and CaMKIIβ. In lamina I, arrowhead indicates a Pax2^−^ calretinin neuron with strong CaMKIIα-LI and weak CaMKIIβ-LI. In lamina IIo, arrowheads indicate two Pax2^+^ calretinin neurons with strong CaMKIIα-LI and weak CaMKIIβ-LI, whereas arrows indicate Pax2^−^ neurons that exhibit considerable CaMKIIα-LI and CaMKIIβ-LI. Dashed line indicates border between laminae I and IIo. In lamina IIi, arrows indicate several neurons with moderate CaMKIIα-LI and CaMKIIβ-LI. Scale bars are 5 µm, valid for all panels

**Fig. 9:**
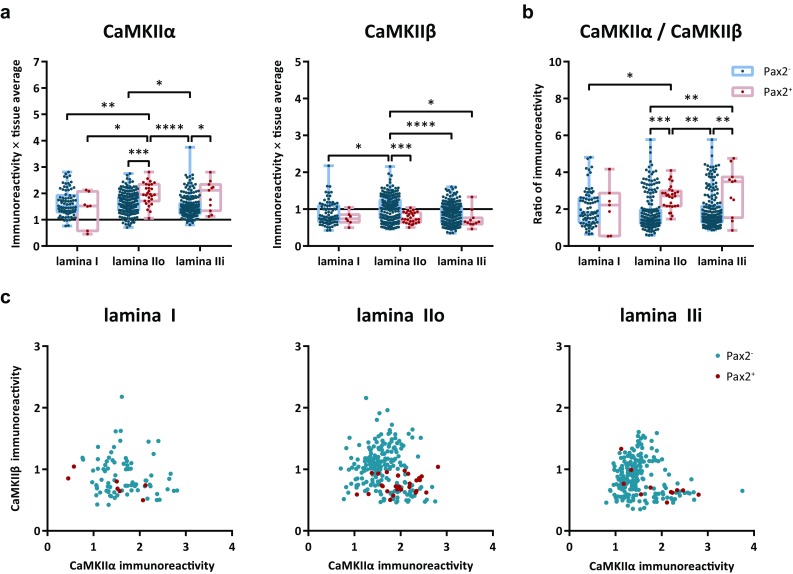
Quantitative analysis of CaMKIIα-LI and CaMKIIβ-LI in calretinin neurons. **a** CaMKIIα-LI and CaMKIIβ-LI normalized against the average intensity over laminae I–III in Pax2^−^ and Pax2^+^ calretinin neurons in laminae I–IIi. Solid horizontal lines indicate average tissue labeling over laminae I–III. **b** Ratio of normalized CaMKIIα-LI over normalized CaMKIIβ-LI in Pax2^−^ and Pax2^+^ calretinin neurons in laminae I–IIi. *p < 0.05; **p < 0.01; ***p < 0.001; ****p < 0.0001; two-way ANOVA followed by Tukey’s post hoc test. **c** Scatterplots of normalized CaMKIIβ-LI versus normalized CaMKIIα-LI in each lamina. Correlations were assessed using Spearman’s correlation. No statistically significant correlations were detected

As the number of sampled Pax2^+^ calretinin neurons was very low in lamina I and lamina IIi, in the following, only Pax2^+^ neurons in lamina IIo will be considered. In Pax2^−^ neurons, CaMKIIα-LI was generally moderate-to-strong (Figs. 8, 9a), similar to the levels found in NeuN/Pax2^−^ neurons in the same lamina (cf Fig. 5a). However, Pax2^+^ calretinin neurons were, on average, more strongly immunolabeled for CaMKIIα than were Pax2^−^ calretinin neurons.

Among Pax2^−^ calretinin neurons, CaMKIIβ-LI was, on average, strongest in lamina IIo. However, Pax2^+^ calretinin neurons exhibited substantially lower levels of CaMKIIβ-LI than did Pax2^−^ calretinin neurons. As expected given the high levels of CaMKIIα-LI and low levels of CaMKIIβ-LI in Pax2^+^ calretinin neurons in lamina IIo, the ratio of CaMKIIα-LI to CaMKIIβ-LI was considerably higher in this population compared to Pax2^−^ calretinin neurons (Fig. 9b). Nevertheless, especially in lamina II, a subpopulation of Pax2^−^/calretinin neurons with high CaMKIIα-LI-to-CaMKIIβ-LI ratio was also evident. No correlation between CaMKIIβ-LI and CaMKIIα-LI was found in any population (Fig. 9c).

### CaMKII isoforms in calbindin D28k neurons

In an initial survey, 7.5% (29/382) of calbindin D28k neurons in laminae I–III was found to co-express Pax2, a fraction which was somewhat higher than the reported proportion of calbindin D28k neurons in the rat dorsal horn that contain GABA (Antal et al. 1991). Nevertheless, the number of Pax2^+^ calbindin D28k neurons in each lamina was low and further quantitative analysis was, therefore, restricted to calbindin D28k neurons that did not express Pax2.

**Fig. 10:**
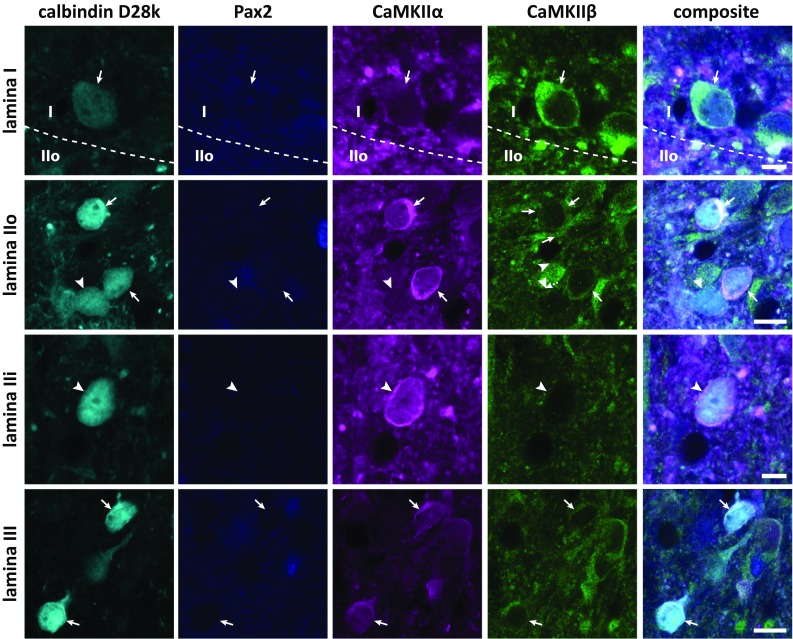
CaMKIIα-LI and CaMKIIβ-LI in calbindin D28k neurons. Shown are examples of Pax2^−^ calbindin D28k neurons with variable CaMKIIα-LI and CaMKIIβ-LI. In lamina I, the arrow indicates a calbindin D28k neuron with moderate CaMKIIα-LI and strong CaMKIIβ-LI. In lamina IIo, arrowhead indicates a neuron with relatively weak CaMKIIα-LI and weak CaMKIIβ-LI, whereas arrows indicate two neurons with strong CaMKIIα-LI and moderate CaMKIIβ-LI. In lamina IIi, arrowhead indicates a neuron with strong CaMKIIα-LI and weak CaMKIIβ-LI. In lamina III, two calbindin D28k neurons moderately labeled for CaMKIIα and CaMKIIβ are indicated by arrows. Scale bars are 5 µm, valid for their respective set of panels

**Fig. 11:**
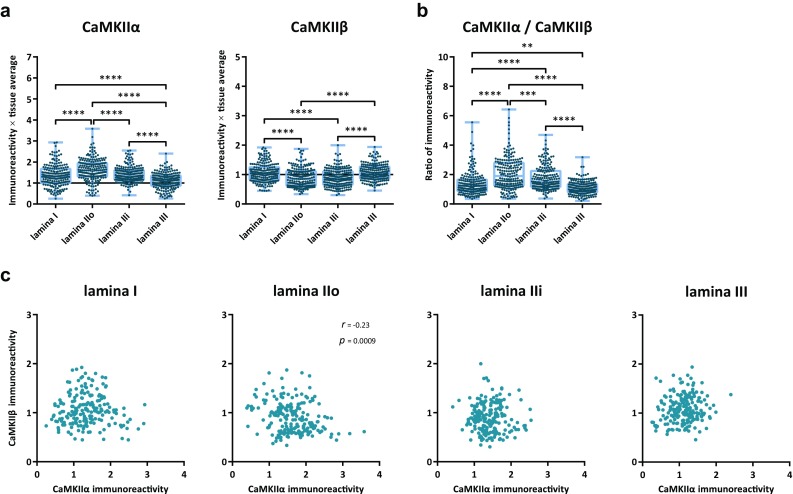
Quantitative analysis of CaMKIIα-LI and CaMKIIβ-LI in calbindin D28k neurons. **a** CaMKIIα-LI and CaMKIIβ-LI normalized against the average intensity over laminae I–III in Pax2^−^ calbindin D28k neurons in laminae I–III. Solid horizontal lines indicate average tissue labeling over laminae I–III. **b** ratio of normalized CaMKIIα-LI over normalized CaMKIIβ-LI in Pax2^−^ calbindin D28k neurons in laminae I–III. **p < 0.01; ***p < 0.001; ****p < 0.0001; one-way ANOVA followed by Tukey’s post hoc test. **c** Scatterplots of normalized CaMKIIβ-LI versus normalized CaMKIIα-LI in each lamina. Correlations were assessed using Spearman’s correlation. A weak negative correlation was found in lamina IIo, but not in other laminae

Calbindin D28k neurons in lamina IIo showed on average higher CaMKIIα-LI than did such neurons in other laminae (Figs. 10, 11). However, although calbindin D28k neurons in lamina III showed the weakest CaMKIIα-LI, the immunolabeling in these neurons relative to tissue average was considerably higher than in NeuN^+^/Pax2^−^ neurons in this lamina (cf Fig. 5). In the case of CaMKIIβ-LI, immunolabeling was stronger in both lamina I and III as compared to either dorsal or ventral lamina II. The levels of CaMKIIβ-LI relative to tissue average were, in all laminae, lower than NeuN^+^/Pax2^−^ neurons in the same laminae (cf. Fig. 5). Median CaMKIIα-LI-to-CaMKIIβ-LI ratio in calbindin D28k neurons in lamina IIo and III was 84 and 98% higher, respectively, than in NeuN^+^/Pax2^−^ neurons in the same laminae.

### CaMKII isoforms at postsynaptic sites

**Fig. 12:**
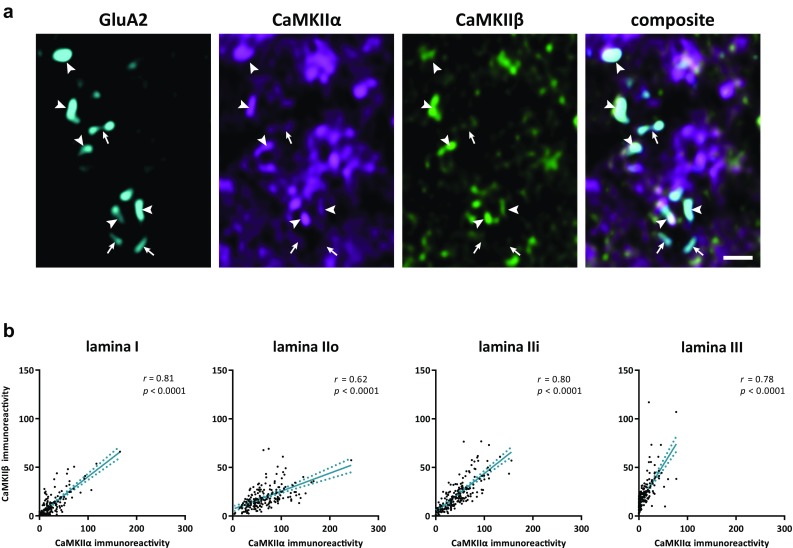
CaMKIIα-LI and CaMKIIβ-LI after pepsin-mediated antigen retrieval to reveal postsynaptic proteins. **a** region of lamina IIi in a spinal cord section immunolabeled for the AMPA receptor subunit GluA2, CaMKIIα, and CaMKIIβ. GluA2 immunolabeling localizes to puncta that presumably correspond to excitatory synapses. CaMKIIα and CaMKIIβ immunolabeling often co-localize in GluA2^+^ puncta at variable levels, although substantial immunolabeling is found also outside such puncta. Arrowheads indicate GluA2^+^ puncta that co-localize with substantial CaMKIIα-LI and CaMKIIβ-LI, whereas arrows indicate GluA2^+^ puncta with weak or no labeling for CaMKIIα or CaMKIIβ. The micrographs are from a single deconvolved optical section obtained using a 63×/1.4 objective. Scale bar, 1 µm valid for all panels. **b** Scatterplots of CaMKIIβ-LI versus CaMKIIα-LI in GluA2^+^ puncta in each lamina. Note the low levels of CaMKIIα-LI in many puncta in lamina III. Correlations were assessed using Spearman’s correlation. Solid and dashed lines indicate linear regression with 95% confidence intervals. Strong positive correlations were found in all laminae

The pool of CaMKII within the postsynaptic density is thought to be pivotal in synaptic plasticity mechanisms (Lisman et al. 2012; Coultrap and Bayer 2012), and it is, therefore, of interest to map this pool in the dorsal horn. However, proteins within the postsynaptic density at glutamatergic synapses are generally not accessible to antibodies with common immunofluorescence procedures. Nevertheless, pepsin-mediated antigen retrieval provides a means to reveal such postsynaptic proteins, generally at the expense of non-synaptic proteins, by degrading surrounding proteins (Larsson et al. 2013; Nagy et al. 2004; Polgár et al. 2008; Watanabe et al. 1998). Pepsin treatment was, therefore, used to investigate postsynaptic CaMKII isoforms at dorsal horn synapses. The AMPA receptor subunit GluA2 was used as a synaptic marker, as this subunit is present at all or essentially all glutamatergic synapses in the dorsal horn (Nagy et al. 2004; Polgár et al. 2008). GluA2 labeling at the surface of pepsin-treated sections through the dorsal horn was punctate and showed a distribution similar to what has been previously described (Larsson et al. 2013; Nagy et al. 2004; Polgár et al. 2008). CaMKIIα and CaMKIIβ immunoreactive puncta often co-localized with GluA2^+^ puncta (Fig. 12a). However, substantial CaMKIIα-LI and CaMKIIβ-LI not associated with GluA2^+^ puncta were also observed, indicating that some pools of non-synaptic CaMKII were resistant to pepsin-mediated degradation under the conditions used here. Moreover, many GluA2^+^ puncta showed weak or undetectable labeling for CaMKIIα and CaMKIIβ. In accordance with the low overall levels of CaMKIIα-LI in lamina III, many GluA2^+^ puncta showed very low or no labeling for CaMKIIα in this lamina. Notably, the intensities of CaMKIIα-LI and CaMKIIβ-LI at GluA2^+^ puncta appeared to co-vary throughout the superficial dorsal horn. Indeed, quantitative analysis showed strong positive correlation between CaMKIIα-LI and CaMKIIβ-LI at GluA2^+^ puncta in all laminae (Fig. 12b).

## Discussion

One of the most salient observations was that CaMKIIα was expressed in all or nearly all neurons in laminae I–III, and at near-similar levels in excitatory and inhibitory neurons. CaMKIIβ, while also ubiquitously expressed in dorsal horn neurons, was found at lower levels in inhibitory neurons as compared to excitatory neurons. Moreover, neuronal populations defined by expression of Ca^2+^-binding proteins showed marked differences in CaMKIIα and CaMKIIβ immunoreactivities.

### Technical considerations

The isoform-specific CaMKII antibodies have been well characterized in the brain (Bachstetter et al. 2014; Ding et al. 2013; Erondu and Kennedy 1985; van Woerden et al. 2009), and their specificity and selectivity in the spinal cord were confirmed here using mice deficient in the respective isoform. Moreover, the extremely low immunoreactivity for either isoform in ependymal cells and microglia in the rat spinal cord further suggests that even the weak immunolabeling found in some neurons reflects specific binding. Nevertheless, it cannot be ruled out that a small proportion of neurons in this study lacked one or both of the isoforms.

The intensity of immunofluorescence may not correlate linearly with antigen density because of a number of confounding factors, including antibody cross-reactivity and variability in epitope accessibility. Thus, it is not possible to deduce, for example, that a cell exhibiting twice as strong immunofluorescence for CaMKIIα as another cell actually possesses twice as many molecules of this isoform. The present observations should, therefore, be interpreted with caution with regard to such comparisons. Nevertheless, the normalized immunofluorescence levels for a given antigen were highly consistent between tissue sections and animals, indicating that the immunolabeling, image acquisition, and analysis procedures were robust. Moreover, additional analysis by manual scoring of NeuN^+^ neurons yielded results consistent with those from direct intensity measurements, further supporting the utility of the latter approach. Thus, it was possible to assess general patterns of immunoreactivity levels in relation to neuronal subpopulations, and, with the caveats noted above, the expression patterns of the respective proteins. Indeed, the quantitative analysis uncovered differences in immunofluorescence of both isoforms that would have been difficult to detect with qualitative methods, including differences in CaMKIIα-to-CaMKIIβ ratio.

Pax2 expression is established as a marker of inhibitory neurons in the mouse and rat dorsal horn (e.g., Cheng et al. 2004; Kardon et al. 2014; Larsson 2017). Thus, the Pax2^+^ neurons in the present study were presumably inhibitory. However, in quadruple immunolabeling experiments, the proportion of Pax2 neurons in lamina III was lower than the proportion of inhibitory neurons in this region, likely because of weak Pax2 expression in some inhibitory neurons. Thus, in these experiments, some inhibitory neurons in lamina III may have been misclassified.

### Excitatory and inhibitory neurons

An unexpected finding of this study was the substantial expression of CaMKIIα also in inhibitory neurons. In the brain, this isoform is believed to be essentially restricted to excitatory neurons (Benson et al. 1992; Sík et al. 1998). Indeed, Benson et al. (1992) reported that also GABA immunoreactive neurons in the dorsal horn lacked CaMKIIα immunoreactivity. This is difficult to reconcile with the present observations, especially as the same monoclonal antibody was used in both studies. However, Benson et al. used Wistar rats fixed with paraformaldehyde and glutaraldehyde, whereas Sprague–Dawley rats fixed without glutaraldehyde were used here; thus, strain and fixation differences could contribute to the observed differences in CaMKIIα expression. Moreover, as GABA levels may be very low in the somata of some inhibitory neurons (Larsson 2017), it is conceivable that an actual presence of CaMKIIα in inhibitory neurons escaped detection in the previous study (Benson et al. 1992).

CaMKIIβ expression was distinctly lower in inhibitory versus excitatory neurons in the superficial dorsal horn. Although the distribution of CaMKIIβ in the rodent spinal cord and elsewhere in the CNS is less well studied than that of CaMKIIα, the isoform is relatively widespread and expressed in some but not all GABAergic neurons (Ochiishi et al. 1994; Terashima et al. 1994; Burgin et al. 1990). The observations of Terashima et al. (1994) on the spinal distribution of CaMKIIβ generally concur with the present study, although they report a somewhat stronger immunolabeling in the superficial dorsal horn compared to the rest of the gray matter. In this regard, the present results are more in line with the transcript distribution in the mouse as described in the Allen Spinal Cord Atlas (Allen Spinal Cord Atlas 2008).

### Parvalbumin neurons

Although some parvalbumin neurons in the rat dorsal horn are excitatory (Antal et al. 1991; Laing et al. 1994), a higher-than-expected proportion of parvalbumin neurons lacking Pax2, and thus classified as presumed excitatory, was found in lamina III. However, as noted above, some of these may have been misclassified because of weak Pax2 expression. Nevertheless, a distinct pattern with respect to CaMKII isoform expression was observed in presumed excitatory parvalbumin neurons. Such neurons in lamina IIi showed strong expression of CaMKIIα and weak expression of CaMKIIβ, whereas presumed excitatory neurons in lamina III showed a lower expression of CaMKIIα but a considerably higher expression of CaMKIIβ. Inhibitory parvalbumin lamina IIi neurons showed moderate levels of CaMKIIα and low levels of CaMKIIβ, whereas inhibitory lamina III parvalbumin neurons showed low levels of both isoforms. Thus, it appears possible to delineate several populations of parvalbumin neurons in the superficial dorsal horn based on transmitter phenotype, location, and CaMKII isoform expression. The relatively low expression of CaMKIIα and CaMKIIβ in inhibitory parvalbumin neurons suggests that these cells are not very susceptible to CaMKII-mediated plasticity.

### Calretinin neurons

About 8% of calretinin neurons in laminae I–II were Pax2^+^, indicating that a small proportion of calretinin neurons in the rat dorsal horn is inhibitory, as is the case in the mouse (Smith et al. 2015; Huang et al. 2010). In lamina II, such neurons showed strong CaMKIIα and weak CaMKIIβ expression. Furthermore, whereas most excitatory calretinin neurons showed moderate levels of both CaMKIIα and CaMKIIβ, a distinct subpopulation of excitatory calretinin neurons in lamina II exhibited a high CaMKIIα-to-CaMKIIβ ratio, similar to inhibitory calretinin neurons. Further studies are needed to determine whether these populations overlap with functionally identified subpopulations of calretinin neuron (Smith et al. 2015).

### Calbindin D28k neurons

Calbindin D28k neurons in lamina IIo were somewhat enriched in CaMKIIα compared to other laminae. Moreover, calbindin D28k neurons in lamina III had higher CaMKIIα levels than unclassified neurons in this lamina. By contrast, CaMKIIβ expression in calbindin D28k neurons was relatively low in all laminae. Indeed, calbindin D28k neurons in laminae IIo and III had substantially higher CaMKIIα-to-CaMKIIβ ratio than other excitatory neurons in these laminae. Thus, although calbindin D28k neurons probably constitute a functionally heterogeneous population (Todd 2017), they may exhibit some degree of functional specificity with regard to CaMKII-mediated processes.

### Postsynaptic CaMKII

The levels of CaMKIIα-LI and CaMKIIβ-LI were positively correlated at GluA2^+^ puncta [presumably corresponding to excitatory synapses (Polgár et al. 2008)] in all superficial laminae in pepsin-treated spinal cord sections. This was in contrast to the soma, which, in most neuronal populations, showed little or no correlation between CaMKIIα-LI and CaMKIIβ-LI. CaMKIIβ is required for targeting the holoenzyme to dendritic spines (Borgesius et al. 2011); therefore, even in cells with low CaMKIIβ expression, postsynaptic pools of CaMKII may be relatively enriched in this isoform, thereby increasing postsynaptic co-variability of CaMKIIα and CaMKIIβ levels. At the same time, as many inhibitory (and some excitatory) neurons in the dorsal horn exhibit few spines (Cordero-Erausquin et al. 2009; Grudt and Perl 2002; Todd and Lewis 1986), synaptic targeting of CaMKII in these neurons may be less dependent on CaMKIIβ.

Many GluA2^+^ puncta showed weak or undetectable immunoreactivity for either CaMKII isoform. It is possible that this reflected a genuine scarcity of CaMKII at these synapses, but it could conceivably also be partly attributed to differential sensitivity to pepsin-mediated degradation of GluA2 and CaMKII, such that CaMKII was largely degraded at some synapses that retained most GluA2 protein.

### Functional and technical implications

In addition to its key role in the early phase long-term potentiation and similar phenomena, CaMKII has been implicated in diverse neuronal processes that may be subject to plasticity, including presynaptic transmitter release, membrane excitability, and excitation–transcription coupling (Hund et al. 2010; Wang 2008; Coultrap and Bayer 2012; Ma et al. 2015; Hell 2014; Lisman et al. 2012). Differences in isoform composition of CaMKII holoenzymes both in the postsynaptic density as well as in the soma and other extrasynaptic compartments may, therefore, impact different forms of neuronal plasticity. Many neurons, including most inhibitory neurons, weakly expressed CaMKIIβ. As this isoform is more sensitive to Ca^2+^ and saturates at lower Ca^2+^ spike frequencies than CaMKIIα, low expression of CaMKIIβ will shift the response curve towards higher Ca^2+^ concentrations and frequencies (Brocke et al. 1999; De Koninck and Schulman 1998); therefore, for instance, neurons with low cytosolic CaMKIIβ levels may require stronger activation to effect mechanisms relying on cytosolic CaMKII, such as excitation–transcription coupling (Ma et al. 2015).

Most parvalbumin neurons as well as many other neurons showed weak labeling for both CaMKIIα and CaMKIIβ. This could reflect a lower propensity for plasticity, but it is also possible that other CaMKII isoforms or CaMKII-independent mechanisms may contribute to plasticity in such neurons. Regardless, the substantial expression of CaMKIIα in many inhibitory neurons suggests that these may also be susceptible to neuronal plasticity dependent on this isoform.

Conditional genetic tools that rely on the *Camk2a* promoter to direct selective protein expression to excitatory neurons are widely used, most commonly in the context of the forebrain but also in the spinal dorsal horn (Lu et al. 2015; Simonetti et al. 2013). However, the expression of CaMKIIα in both excitatory and inhibitory neurons in the superficial dorsal horn suggests that caution should be exercised when using such tools, especially in the dorsal horn, where they may not be valid for selective targeting of excitatory neurons.

## Conclusions

This mapping of CaMKIIα and CaMKIIβ in the rat superficial dorsal horn revealed extensive co-localization in all or nearly all excitatory and inhibitory neurons. Moreover, different populations of dorsal horn neuron defined by transmitter phenotype, calcium-binding proteins, and location differed in their pattern of expression of each isoform. This implicates that whereas CaMKII-mediated signaling and plasticity may be ubiquitous in the superficial dorsal horn, neuronal populations exhibit differences in the characteristics of such mechanisms.
